# Extract of *Pogostemon cablin* Possesses Potent Anticancer Activity against Colorectal Cancer Cells In Vitro and In Vivo

**DOI:** 10.1155/2020/9758156

**Published:** 2020-09-09

**Authors:** Ju-Huei Chien, Shan-Chih Lee, Kai-Fu Chang, Xiao-Fan Huang, Yi-Ting Chen, Nu-Man Tsai

**Affiliations:** ^1^Department of Laboratory Medicine, Taichung Tzu-Chi Hospital, Buddhist Tzu-Chi Medical Foundation, Taichung 42743, Taiwan; ^2^Department of Medical Laboratory Science and Biotechnology, Central Taiwan University of Science and Technology, Taichung 40601, Taiwan; ^3^Department of Medical Imaging and Radiological Sciences, Chung Shan Medical University, Taichung 40201, Taiwan; ^4^Department of Medical Imaging, Chung Shan Medical University Hospital, Taichung 40201, Taiwan; ^5^Institute of Medicine, Chung Shan Medical University, Taichung 40201, Taiwan; ^6^Department of Medical Laboratory and Biotechnology, Chung Shan Medical University, Taichung 40201, Taiwan; ^7^Clinical Laboratory, Chung Shan Medical University Hospital, Taichung 40201, Taiwan

## Abstract

*Pogostemon cablin* (PCa), an herb used in traditional Chinese medicine, is routinely used in the amelioration of different types of gastrointestinal discomfort. However, the mechanisms underlying the cancer suppression activity of PCa in colorectal cancer (CRC) cells have yet to be clarified. The aim of this study was to investigate the anticancer effects of PCa, specifically the induction of apoptosis in CRC cells. The growth inhibition curve of CRC cells following exposure to PCa was detected by an MTT assay. Moreover, PCa combined with 5-FU revealed a synergic effect of decreased cell viability. PCa inhibited cell proliferation and induced cell cycle arrest at the G_0_/G_1_ phase and cell apoptosis through regulation of associated protein expression. An in vivo study showed that PCa suppressed the growth of CRC via induction of cell apoptosis with no significant change in body weight or organ histology. Our results demonstrated that PCa inhibits the growth of CRC cells and induces apoptosis in vitro and in vivo, which suggests the potential applicability of PCa as an anticancer agent.

## 1. Introduction

Colorectal cancer (CRC) is one of the most common malignant cancer types. It has a high incidence in developed countries [1]. In 2018, around 1.85 million cases were diagnosed, and 880,792 deaths occurred from CRC worldwide [2]. The 5-year survival rate is around 90% for patients diagnosed with early-stage cancer; however, the 5-year survival rate drops to only 10% if there is distant metastasis at the time of diagnosis. The first line of treatment is surgery with curative potential for CRC patients [3], which is performed in 75–80% of newly diagnosed patients with localized or regional tumors; however, around 50% of them will develop a recurrence after surgery [4, 5]. Chemotherapy is used as adjuvant therapy for prevention of local recurrences or distant metastases. 5‐Fluorouracil (5‐FU), the first‐line chemotherapeutic drug of CRC, is an antimetabolite agent that has poor efficacy against CRC, with only 10% to 15% of patients responding [6, 7], due to the limitations of severe side effects and the development of drug resistance [8, 9]. Therefore, it is necessary to develop novel therapeutic agents or strategies for CRC therapy.

Natural products have been recognized as a source of bioactive components, and they are used in both traditional health care systems and new drugs development [10]. More novel natural products with high bioefficiency and low toxicity have been discovered, and they have the potential for development into new drugs that are not derived from fully synthetic or combined synthetic compounds [11, 12]. Many reports have revealed that approximately half of all approved drugs were developed from natural plants, such as paclitaxel, vinblastine, and etoposide [13–15]. In cancer therapy, natural products or their derivatives not only induce cell apoptosis but also act against the resistance of cancer cells to chemotherapeutic drugs through regulation of cell apoptosis or combination treatment [16–18]. Therefore, natural products have been introduced as a potential strategy for cancer treatment and prevention.

*Pogostemon cablin* (PCa) is an herb that has been used in traditional Chinese medicine for hundreds of years to clinically ameliorate gastrointestinal diseases and dispel dampness and superficies syndrome [19], and it has been used as a complementary anticancer agent for a few years [20]. The major compounds in PCa include patchouli alcohol, azulene, *α*-guaiene, and seychellene [19, 21]. The biological activities of PCa have been widely reported, including anti-inflammatory, antioxidative, antimicrobial, analgesic, antiplatelet, antithrombotic, antidepressant, and antiemetic effects [19, 22, 23]. In a previous study, *Pogostemon cablin* aqueous extract could induce apoptosis in endometrial cancer cells in vitro [24]. However, the anticancer activity of PCa is yet to be clarified in CRC in vitro and in vivo. Here, we investigated the activities and mechanisms of PCa against CRC cells both in vitro and in vivo.

## 2. Materials and Methods

### 2.1. Chemicals and Reagents

MTT (3-(4,5-dimethylthiazol-2-yl)-2,5-diphenyltetrazolium bromide), dimethyl sulfoxide (DMSO), 5-fluorouracil (5-FU), propidium iodide (PI), and RNase were obtained from Sigma-Aldrich (St. Louis, CA, USA). Dulbecco's modified Eagle medium (DMEM) and Roswell Park Memorial Institute Medium-1640 (RPMI), fetal bovine serum (FBS), HEPES, pyruvate, and penicillin–streptomycin (S/P) were from Gibco-BRL (Grand Island, NY, USA). The leaves of *Pogostemon cablin* were from Republik Indonesia, with confirmation of identification by Professor Han-Ching Lin. The small-scale extraction was prepared in our laboratory, and the conditions were described as follows. The leaves of *Pogostemon cablin* (500 g) were dehydrated, crushed, and placed in a 2 L steam distillation steel apparatus unit. Extraction was performed by hydrodistillation with a flow rate of approximately 7.2 ml/min at 100°C for 100 min, and the yields were about 2.09%. The large scale of PCa extract was entrusted by Phoenix (New Jersey, USA).

### 2.2. Cell Culture and Treatment

Human colorectal adenocarcinoma cells (HT-29), mouse colon carcinoma cells (CT26), mouse vascular endothelial cells (SVEC), and canine kidney epithelial cells (MDCK) were purchased from the Food Industry Research and Development Institute (Hsinchu, ROC). Cells were cultured in DMEM (HT-29, SVEC, and MDCK cells) and RPMI (CT26 cells) supplemented with 10% FBS, HEPES, pyruvate, and S/P and maintained at 37°C in a humidified incubator with 5% CO_2_. The status of TP53, BRAF, KRAS, and PIK3CA in the HT-29 cells were mutated, detected using automated extraction of nucleic acids (AccuBioMed Co., Ltd., Taipei, Taiwan) and FemtoPath Human Primer Sets (HongJing Biotech, Taipei, Taiwan). The stock solution (50 mg/ml) of PCa was prepared in dimethylsulfoxide (DMSO) immediately before use, and the cells were treated with PCa at different times (30 *μ*g/ml for 0–48 h) and at different dosages (0–45 *μ*g/ml for 24 h).

### 2.3. Cytotoxicity Assay

The cell viability was detected by an MTT assay. Cells at a density of 5 × 10^3^ cells/well were cultured in 96-well culture plates overnight and then treated with different concentrations of PCa or 5-FU for 24, 48, and 72 h. Then, the culture medium was replaced with MTT solution (400 *μ*g/ml, Sigma) and incubated for 6–8 h. Later, the formazan crystals were dissolved in 50 *μ*l DMSO, and the absorbance intensity was measured with a microplate reader (Molecular Device/Spec384) at 550 nm. Percentage of cell survival was calculated using the following formula: cell viability (%) = intensity (treated cell)/intensity (control cell) × 100%.

### 2.4. Determination of Combination Effect

HT-29 cells were seeded in 96-well culture plates and treated with PCa (0, 10, 20, 40, or 80 *μ*g/ml) combined with 1.5 *μ*g/ml 5-FU or 5-FU (0, 1, 2, 4, or 8 *μ*g/ml) combined with 30 *μ*g/ml PCa for 48 h, and then we detected the cell viability by an MTT assay. The value of the combination index (CI), determined synergism (CI < 1), additive effect (CI = 1), and antagonism (CI > 1) were calculated by the following: [IC_50_ (drug A + B)/IC_50_ (drug A)] + [IC_50_ (drug A + B)/IC_50_ (drug B)] [25].

### 2.5. Detection of the Cell Cycle Stage

Cell cycle progression was analyzed by flow cytometry through the determination of DNA content in cell nuclei stained with propidium iodide (PI). The treated cells were harvested and stained by incubation in PBS containing 40 *μ*g/ml PI and 100 *μ*g/ml RNase for 30 minutes at 4°C. The fractions of the cells in the G1, S, and G2 phases were measured and analyzed using a FACScan (Beckton Dickinson, USA) and FlowJo software (Tree Star, San Carlos, CA, USA).

### 2.6. TUNEL Assay

Apoptosis was determined by using an In Situ Cell Death Detection Kit (TUNEL), POD (Roche, Mannheim, Germany) according to the manufacturer's instructions. Cells smeared on silane-coated slides or deparaffinized tissue sections were incubated with 3% H_2_O_2_ in methanol and 0.1% Triton X-100 in 0.1% sodium citrate buffer on ice to increase the cells' permeability. Cells or tissues were incubated with a TUNEL detection kit for 2 h at 37°C, counterstained with PI staining, and observed under a fluorescence microscope (ZEISS AXioskop 2) at 400× magnification.

### 2.7. Western Blot Analysis

The procedures of protein extraction and Western blotting were performed as previously described [26]. Specific primary antibodies used in this study included those against p53, p-p53, p21, p-Rb, PCNA, CDK2, CDK4, cyclin A, cyclin B1, cyclin D1, FAS, Caspase-8, Bax, Caspase-9, Caspase-3, MMP2, and MMP9 (Santa Cruz, CA, USA); *β*-actin (iReal Biotechnology Co., Ltd., Hsinchu, Taiwan). The antigen–antibody complexes were detected by a chemiluminescence imaging analyzer (GE LAS-4000, GE Healthcare Life Sciences, NJ, USA), and the signal intensity of each band was determined by using ImageJ software 1.47t (National Institutes of Health, Bethesda, MD, USA).

### 2.8. Animal Study

BALB/c female mice (10–12 weeks, 19–23 g) were purchased from the National Laboratory Animal Center (Taipei, ROC) and housed, 6 per cage, in a laminar airflow room and maintained on laboratory standard experimental conditions (relative humidity 55–60%, dark/light cycle 12/12 h, and free access to balanced diet and water at a temperature of 25 ± 1°C). Mice were adapted to laboratory conditions for a week before experimentation. The research was performed in Chung Shan Medical University (CSMU) following the Guide for the Care and Use of Laboratory Animals and approved by the Institutional Animal Care and Use Committee (IACUC) of CSMU (Approval no. CSMU-IACUC-1543). The mice (*n* = 10) were subcutaneously injected with 1 × 10^6^ CT26 cells in PBS into the flank. Seven days later, the tumor-bearing mice were randomly divided into 2 groups: (1) vehicle (*n* = 4) were treated with 5% DMSO in PBS by subcutaneous injection and (2) PCa groups (*n* = 6) were treated with 200 mg/kg PCa by subcutaneous injection once every 2 days for 40 days. Tumor size and body weight were recorded every 2 days. When the tumor volume was greater than 1500 mm^3^ (*L* × *H* × *W* × *π*/6 mm^3^), mice were sacrificed using carbon dioxide. The organs, including the liver, kidney, and intestine, were collected, fixed with 4% neutral formalin, embedded in paraffin, and cut for HE staining analysis. Sections were deparaffinized, rehydrated, stained with hematoxylin and eosin (Muto Pure Chemicals, Tokyo, Japan), observed, and photographed under a bright-field microscope.

### 2.9. Statistical Analysis

Data were analyzed using Student's *t*-test, and the survival rate was analyzed using the Kaplan–Meier method to determine the statistical significance of differences (*P* values of <0.05). The results are expressed as the mean ± standard deviation (SD) for the in vitro data or standard error (SE) for the in vivo data.

## 3. Results

### 3.1. PCa Inhibited the Growth of CRC Cells In Vitro

**Figure 1:**
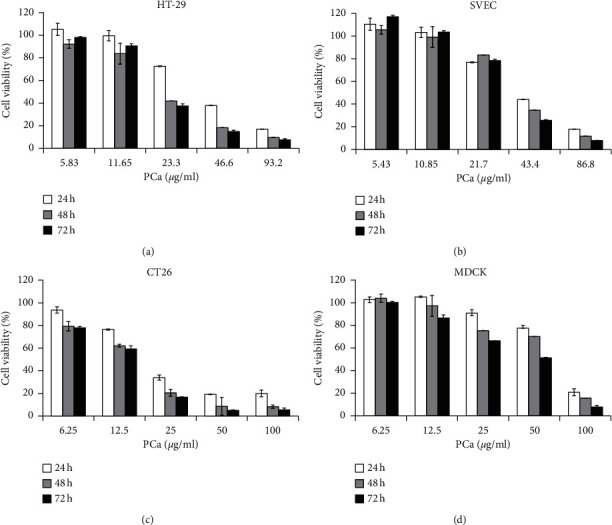
Effect of PCa on cell survival and proliferation of CRC cells. Cell viability was determined by using an MTT assay after treatment for 24, 48, or 72 h with increasing concentrations of PCa. (a) HT-29, (b) CT26, (c) SVEC, and (d) MDCK cells. The percentage of cell viability was normalized to the control (100%). The results are expressed as mean ± SD.

The PCa was diluted in hexane (1 : 50), analyzed with a GC-MS, and identified via comparison with mass spectra from the literature and the NIST and Wiley Library databases. The major components smaller than 500 daltons in the PCa included azulene (21.81%), *α*-guaiene (18.85%), patchouli alcohol (18.16%), *α*-patchoulene (11.14%), *γ*-gurjunene (9.31%), and others (data not shown). MTT assays were conducted to examine the cytotoxicity of PCa against HT-29 and CT26 cells. There was a dose-dependent cytotoxic effect of PCa treatment for 24–48 h against CRC cells (Figures 1(a) and 1(b)). However, the cytotoxicity against the normal cells (SVEC and MDCK) was lower than that for the CRC cells (Figures 1(c) and 1(d)). The IC_50_ values of HT-29, CT26, SVEC, and MDCK were 21.04 ± 0.68, 15.46 ± 1.28, 40.35 ± 2.91, and 68.55 ± 0.28 *μ*g/ml for 48 h, respectively (Table 1). These results suggest that PCa inhibited the growth of CRC cells in a dose-dependent manner but was less cytotoxic to normal cells.

### 3.2. PCa Combined with 5-FU Synergistically Inhibited CRC Cells

**Figure 2:**
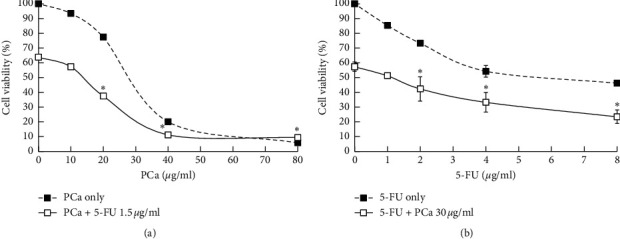
PCa and 5-FU synergistically inhibited the growth of HT-29 cells in vitro. HT-29 cells were treated with (a) PCa (0, 10, 20, 40, or 80 *μ*g/ml) and/or 5-FU (1.5 *μ*g/ml), (b) 5-FU (0, 1 2, 4, or 8 *μ*g/ml) and/or PCa (30 *μ*g/ml) for 48 h, and then we measured cell viability by using an MTT assay. PCa and 5-FU revealed a synergistic effect (CI value < 1). ^*∗*^Compared with PCa or 5-FU only vs. the combination group (*P* < 0.05).

To determine the effect of a combination of PCa and 5-FU, HT-29 cells were treated with PCa (10, 20, 40, or 80 *μ*g/ml) combined with 5-FU 1.5 *μ*g/ml or 5-FU (1, 2, 4, or 8 *μ*g/ml) combined with PCa 30 *μ*g/ml. The cell viability was measured by using an MTT assay after treatment for 48 h. As shown in Figure 2, the viability of the drugs combination group was not only lower than that of the PCa only but also than that of the 5-FU only, and the combination index (CI) was 0.65, indicating a synergistic effect (CI < 1). These results pointed to a synergistic effect of the activity of PCa and 5-FU against CRC cells.

### 3.3. Effect of PCa on Cell Cycle Arrest in HT-29 Cells

**Figure 3:**
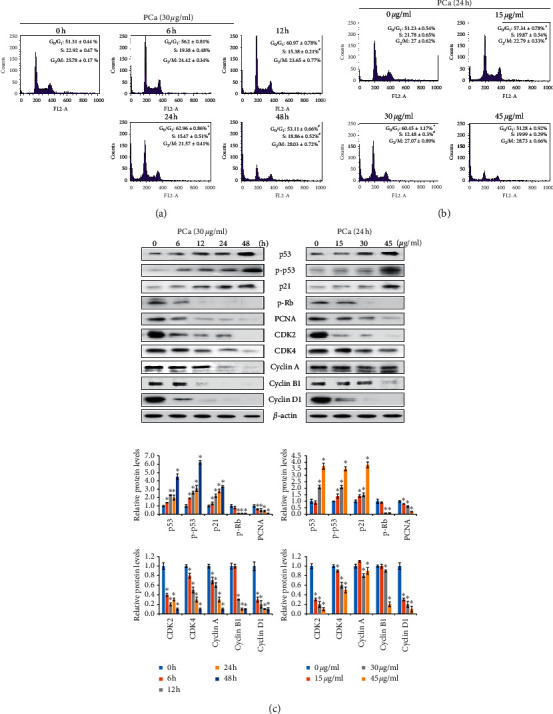
PCa induced G_0_/G_1_ phase cell cycle arrest by decreasing CDK4 and cyclin D1 expression. Cell cycle distribution (G_0_/G_1_, S, G_2_/M phases) of HT-29 cells after treatment with 30 *μ*g/ml PCa for 0–48 h (a); 15, 30, or 45 *μ*g/ml PCa for 24 h (b) was detected and analyzed by flow cytometry and FlowJo software. The results are shown as mean ± SD. ^*∗*^*P* < 0.05 versus control group with a significant increase. ^#^*P* < 0.05 versus control group with a significant decrease. (c) Western blot showing the upregulation of the proteins p53 and p21, whereas downregulation of the proteins CDK4 and cyclin D1 was observed in HT-29 cells.

The PCa-induced decrease in cell viability could have been a result of decreased proliferation. Next, to investigate whether PCa induced cell cycle arrest, flow cytometry was used to analyze the distribution of the cells in the cell cycle. After the HT-29 cells were treated with PCa for different times or at different doses, the percentage of cells in the G_0_/G_1_ phase was increased in doses of 15 and 30 *μ*g/ml (Figures 3(a) and 3(b)), indicating PCa-induced cell cycle arrest in the G_0_/G_1_ phase. The protein expression of p53, p-p53, and p21 was increased, but that of p-Rb, PCNA, CDK2, CDK4, cyclin A, cyclin B1, and cyclin D1 was decreased in the PCa-treated cells (Figure 3(c)). Therefore, these results suggested that PCa-induced growth inhibition of CRC cells was associated with cell cycle arrest via the regulation of cell cycle regulators.

### 3.4. PCa Promotes Cell Apoptosis in CRC Cells

**Figure 4:**
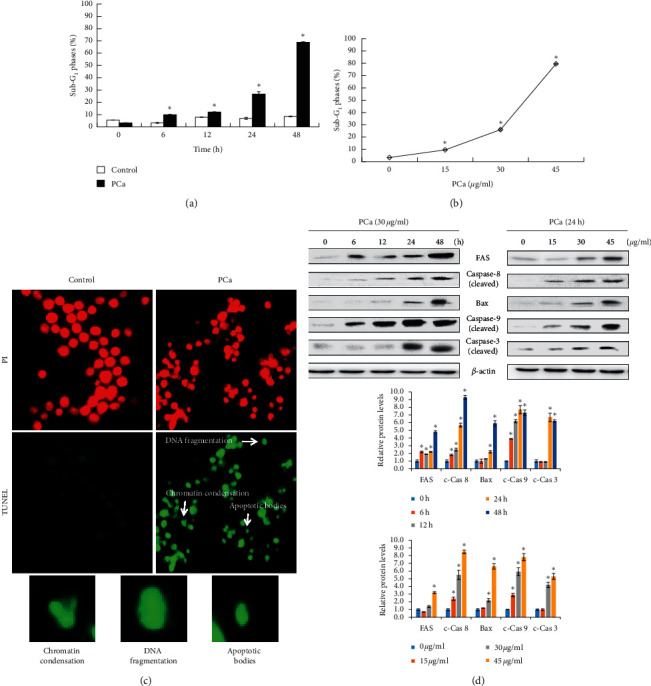
PCa triggered cell apoptosis through extrinsic and intrinsic pathways in HT-29 cells. (a, b) HT-29 cells were treated with PCa and analyzed for SubG_1_ phase by flow cytometry. Data are expressed as mean ± SD. ^*∗*^*P* < 0.05 versus control group with a significant increase. (c) The typical morphology of cell apoptosis, such as chromatin condensation, DNA fragmentation, and apoptosis body, was observed after PCa treatment for 48 h by using TUNEL assays. (d) Determination of the apoptosis pathway by Western blots of PCa-treated cells.

The percentage of cells in the SubG_1_ phase was significantly increased in PCa-treated cells compared with the control as determined by flow cytometry (Figures 4(a) and 4(b)). Next, to determine the effect of PCa on apoptosis, PCa-treated cells were subjected to a TUNEL assay. The PCa-treated cells had positive TUNEL results and exhibited morphology consistent with cell apoptosis, such as chromatin condensation, DNA fragmentation, and apoptosis bodies (Figure 4(c)). In the apoptotic molecular analysis, PCa promoted the expression and activation of Fas/caspase-8, Bax/caspase-9, and caspase-3 (Figure 4(d)). All of these results suggested that PCa induced cell apoptosis through the activation of caspases in CRC cells.

### 3.5. PCa Reduced Protein Expression of Factors Involved in Metastasis

**Figure 5:**
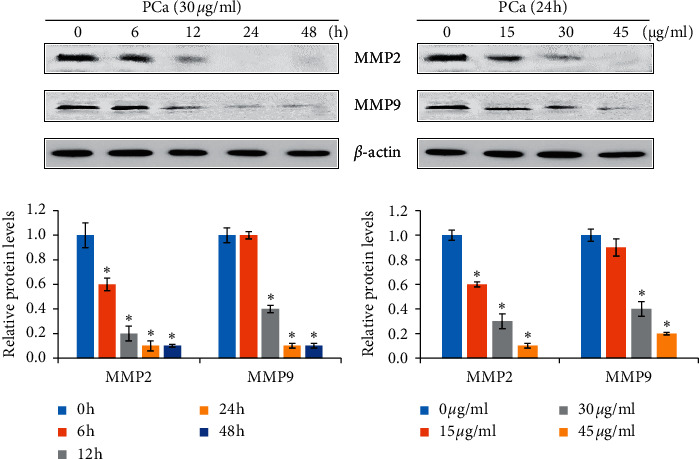
The molecules involved in metastasis were affected by PCa treatment of HT-29 cells. Cells were treated with 30 *μ*g/ml PCa for 0, 6, 12, 24, or 48 h; 15, 30, or 45 *μ*g/ml PCa for 24 h, and the protein expression of MMP2 and MMP9 was analyzed by Western blotting. Protein bands were normalized using the corresponding *β*-actin to calculate the band density. MMP: matrix metallopeptidase.

Previous studies indicated the fact that high metastasis resulted in a poor prognosis of CRC patients. Therefore, we investigated whether PCa regulated metastasis-associated protein expression. Western blotting showed that PCa reduced the expression of the metastasis proteins MMP2 and MMP9 (Figure 5). These results indicated that PCa has a potential effect on antimetastasis of CRC cells.

### 3.6. PCa Suppressed the Growth of CRC in Tumor-Bearing Mice

**Figure 6:**
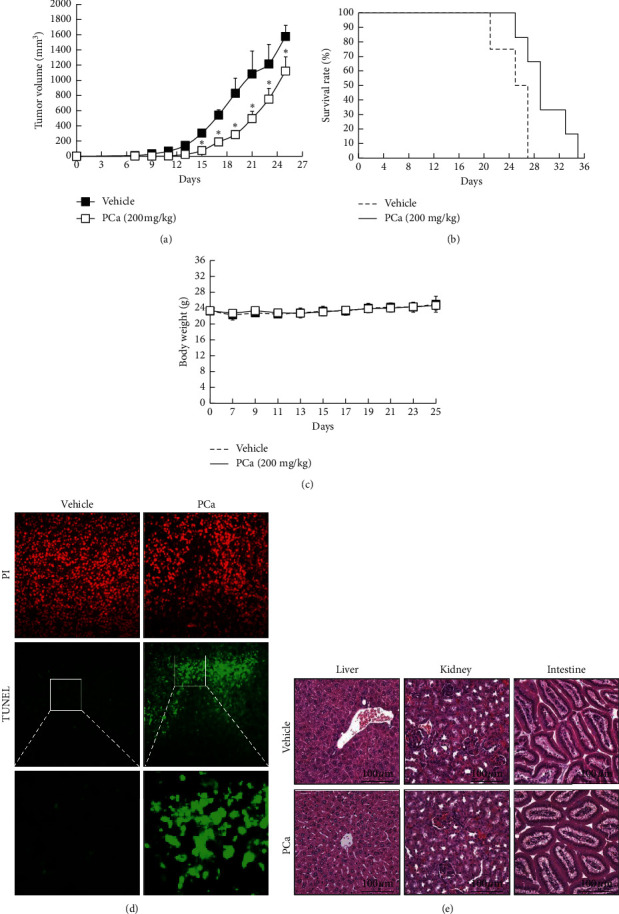
PCa suppressed tumor growth via induction of cell apoptosis in vivo. Mice were subcutaneously implanted with CT26 cells and treated (s.c.) with 200 mg/kg PCa once every two days for 40 days. Tumor volume (a), survival (b), and body weight (c) were recorded once every two days, and mice were sacrificed when the volume of the tumor exceeded 1500 mm^3^. Results are expressed as mean ± SD. ^*∗*^*P* < 0.05 versus the vehicle group. (d) Cell apoptosis was determined through tissue TUNEL assays. (e) HE staining of the organs, including liver, kidney, and intestine, was used for assessing toxicity.

To examine the antitumor effects of PCa in vivo, a therapeutic animal model was established by transplanting CT26 cells into BALB/c female mice. As shown in Figures 6(a) and 6(b), the tumor volume in the PCa group (1121.07 ± 187.65 mm^3^) was smaller than that in the vehicle group (1577.33 ± 146.49 mm^3^) at day 25, and there was a prolonged survival time (from 27 to 35 days), indicating that PCa significantly inhibited the growth of CT26 tumors. In tissue TUNEL assays, there were obvious positive results in the PCa groups (Figure 6(c)). Moreover, there was no significant difference between the vehicle and PCa groups for body weight or organ histology, including the liver, kidney, and intestine (Figures 6(d) and 6(e). In short, these results suggested that PCa suppressed tumor growth via induction of cell apoptosis, with no significant side effects on the mice.

## 4. Discussion

Colorectal cancer is the most common type of gastrointestinal cancers and is a leading cause of cancer-related deaths among men and women worldwide [27]. Severe adverse effects and limitations in the therapeutic efficacy of conventional clinical drugs for cancer treatment have led to the widespread application of complementary and alternative medicine [28]. Among these treatments, traditional medicines with demonstrable bioactivity against cancer provide atleast 250 preferred choices [29]. In this study, we selected *Pogostemon cablin* to investigate its anti-CRC effect in vitro and in vivo. We used two CRC cells, namely, HT-29 (human colorectal adenocarcinoma) and CT26 cells (mouse colon carcinoma), to perform different experiments. HT-29 cells were used to examine the in vitro anticancer mechanism of PCa. The results demonstrated that PCa induced cell cycle arrest at G_0_/G_1_ phase via p53 and p21 upregulation and CDKs/cyclins downregulation, then promoting cell apoptosis via activation of extrinsic and intrinsic cell apoptosis pathway. CT26 cells were used to evaluate the therapeutic effect on subcutaneous tumor in BALB/c mice model. The results suggested that PCa suppressed CRC growth and induced cell apoptosis in vivo, where anticancer mechanisms were consistent with in vitro assays. These findings highlight the anticancer potential of PCa against colorectal carcinoma.

Cell proliferation is controlled by cell cycle progression, which is divided into four nonoverlapping phases, G_1_, S, G_2_, and M, with a strong regulatory process, and it has checkpoints that cause cell cycle arrest [30]. Our study demonstrated that PCa induced cell cycle arrest at the G_0_/G_1_ phase in CRC cells, leading to a decrease in cell viability in time- and dose-dependent manners. p53 activation exerts tumor suppression through promoting the transcription of target genes such as p21 and p27 [31]. p21 belongs to the Cip/Kip family of CDK inhibitors that bind to cyclin/CDK complexes to inhibit activity, thereby subsequently arresting the cell cycle. CDK/cyclins are divided into regulators of different cell cycle phases, including G_0_/G_1_ phase: cyclin D and CDK4/6; S phase: cyclin E and CDK2; and G_2_/M phase: cyclin A, cyclin B1, and CDK1/2 [32]. In this study, treatment with PCa resulted in upregulation of p53 and p21 and downregulation of CDK2, CDK4, cyclin A, cyclin B1, and cyclin D1 proteins in time- and dose-dependent manners. Although the cell cycle assessment identified G_0_/G_1_ phase arrest, overall, the cell cycle-related proteins decreased following exposure to PCa. In addition, downregulated expression of PCNA protein by PCa implied a decreased proliferation ability. These results suggest that PCa inhibits cell proliferation of CRC cells via cell cycle arrest, especially arrest at the G_0_/G_1_ phase, and upregulation of p53 and p21 proteins expression.

Apoptosis is a type of programmed cell death with specific morphological features, such as an increase of the cell population arrested in the sub-G_1_ phase, chromatin condensation, DNA fragmentation, and apoptotic bodies, which is associated with activation of caspase cascades [33]. We found that PCa treatment significantly increased the accumulation of cells in the sub-G1 phase and induced cell apoptosis with typical apoptotic morphological features in vitro and in vivo by performing TUNEL assays. Apoptosis is induced via the regulation of caspase cascades, and caspases are divided into the initiators, including the extrinsic pathway (caspases-2, -8, and -10) and the intrinsic pathway (caspase-9); and the effector (caspases-3, -6, and -7) caspases. Caspase-3 is a key enzyme in the production of cysteinyl aspartate and is a central apoptosis regulator due to apoptosis being initiated by most triggers through the caspase-3-mediated pathway [33]. In this study, we showed that the levels of Fas, Bax, and caspases-3, -8, and -9 increased in CRC cells following PCa treatment. These results suggest that PCa induces cell apoptosis via induction of the extrinsic and intrinsic apoptosis pathways in CRC cells.

Previous studies demonstrated that *Pogostemon* species contained anticancer activity. An extract of *Pogostemon benghalensis* Linn. showed anticancer activity on Ehrlich ascites carcinoma in tumor-bearing mice [34]. *Pogostemon cablin* aqueous extract induced apoptosis in endometrial cancer (Ishikawa) cells [24]. Petroleum ether and chloroform leaf extracts of *Pogostemon quadrifolius* (Benth.) inhibited the growth of human colorectal (Caco-2), cervix (HeLa), monocytic leukemia (THP-1), breast (MCF-7), and leukemic T cell lymphoblast (Jurkat E6-1) cancer cells [35]. *Pogostemon deccanensis* essential oils have antiproliferation activity in the mouse cancer cell line B16F1 [36]. In this study, we demonstrated that PCa inhibited cell growth and induced cell apoptosis of CRC cells in vitro and in vivo.

Furthermore, the components in PCa were analyzed by GC-MS and identified by comparison with mass spectra from the literature and the NIST and Wiley Library databases. The major compounds smaller than 500 daltons in the PCa included azulene (21.81%), *α*-guaiene (18.85%), patchouli alcohol (18.16%), *α*-patchoulene (11.14%), *γ*-gurjunene (9.31%), and others. Recent studies showed that azulene had antiproliferation activity against MCF7 breast cancer cells and DU145 prostate cancer cells, and different substituted azulene derivatives have been developed [37]. Another component, *α*-guaiene, was found to be responsible for most of the cytotoxic activity of *Bulnesia sarmientoi* against A549 and H661 lung cancer cells [38]. Exposure of HCT116 and SW480 colorectal cancer cells to patchouli alcohol activated p21 expression and inhibited the expression of cyclin D1 and CDK4 and induced cell apoptosis and cell cycle arrest in A549 lung cancer cells in vitro and in vivo via the EGFR-MAPK pathway [39, 40]. A hexane fraction of guava leaves (*Psidium guajava* L.), which contained 3.76% *α*-patchoulene, inhibited the AKT/mTOR/S6K1 signaling pathway and induced apoptosis in prostate cancer cells [41]. The oleoresin extract of *Dipterocarpus alatus* (contained 3.14% *γ*-gurjunene) showed cytotoxicity against human leukemic U937 cells [42]. These studies indicated that PCa contains various compounds with anticancer activity, suggesting that PCa has a high potential for development into an anticancer agent or adjuvant treatment in colorectal cancer therapy in the future.

## 5. Conclusions

PCa suppressed the cell growth of CRC in vitro and in vivo and, combined with the clinical drug 5-FU, synergistically inhibited proliferation of CRC cells. PCa induced cell cycle arrest at the G_0_/G_1_ phase and upregulated the expression of p53 and p21 and downregulated CDK4 and cyclin D1 proteins. PCa induces cell apoptosis via induction of the extrinsic (Fas/caspase-8) and intrinsic (Bax/caspase-9) apoptosis pathway in CRC cells. Moreover, PCa downregulated metastasis-associated proteins (MMP2/MMP9) expression. Thus, these results indicate that PCa should be investigated further for its potential contributions to CRC treatment.

## Figures and Tables

**Table 1:** The IC_50_ values of PCa against CRC and normal cells.

Cell line	Tumor type	PCa	5-FU
HT-29	Human colorectal adenocarcinoma	21.04 ± 0.68^a,b^	8.60 ± 1.52
CT26	Mouse colon carcinoma	15.46 ± 1.28^a,b^	0.3 ± 0.01
SVEC	Mouse endothelia cell	40.35 ± 2.91^a^	<6.25
MDCK	Canine kidney epithelial cell	68.55 ± 0.28^a^	12.30 ± 1.53

*Note*. Values are expressed as mean ± SD (*μ*g/ml) at 48 h. ^a^A significant difference between the PCa group compared with the 5-FU group. ^b^A significant difference between the CRC cells compared with normal cells.

## Data Availability

All data generated or analyzed during this study are included in this published article.
